# "The Hospital" Nursing Mirror

**Published:** 1898-01-29

**Authors:** 


"The Hospital" Nursing Mirror.

Being the Nursing Section of "The Hospital."

[Contributions for this Section of "The Hospital" should be addressed to the Editor, The Hospital, 28 & 29, Southampton Street, Strand, London, W.C., and should have the word "Nursing" plainly written in left-hand top corner of the envelope.]

### News from the Nursing World.

### THE PRINCESS CHRISTIAN'S VISIT TO HAMPSTEAD.

On March 5th the Princess Christian will visit Hampstead for the purpose of opening the two beds endowed in perpetuity in memory of the Diamond Jubilee at the Hampstead Hospital and Nursing Institution.

### THE PRINCESS LOUISE AT BIRKENHEAD.

One of the pleasant duties performed by the Princess Louise on her visit to Liverpool and Birkenhead was the reception of purses from about fifty children on behalf of the endowment fund of the nurses' home. The ceremony was arranged in the upper room of Messrs. Della Robbia's pottery factory in Price Street, Birkenhead. It was prettily decorated, and the children for the most part were dressed in white. The Mayor's daughter, Miss Mason, presented the Princess with a bouquet, and the purses contained £300, in sums varying from £5 to £20. The nurses' home itself was Birkenhead's memorial of her Majesty's long reign.

### A DEVOTED WIFE.

Prince Oscar of Sweden has undertaken missionary work in West Africa, and his wife, the Countess of Wisborg, is at present training as a nurse in London in order that she may help him.

### NURSING NOTES FROM GUY'S HOSPITAL.

Miss Morrah, Sister Astley Cooper, has resigned owing to private reasons. Her duties at Guy's Hospital have been undertaken by Miss Leng, who will be succeeded in her work in the Mary ward by Miss Hodges, the present senior night sister. Miss Cawley, sister of the Martha ward, has also resigned for family reasons. An addition of 30 nurses to the staff has been required to carry into effect the new off-duty regulations, and this number of probationers will begin work on January 31st. The senior probationers will for the future be distinguished by a badge, consisting of a narrow white band, placed on the sleeve four inches from the wrist.

### TYPHOID AT MAIDSTONE.

We regret to learn that typhoid is not yet extinct at Maidstone. Seven fresh cases have occurred during the last fortnight, amongst which is that of Nurse Wilkinson, who has been employed there in nursing during the epidemic.

### NURSES VINDICATED.

The nurses of the St. George's-in-the-East Infirmary have been completely cleared of the very grave charge lately made against them by the relatives of a patient. It appears that a child recovering from measles died suddenly of convulsions, and after its burial the parents became possessed of the idea that it had been strangled by the tie, used to prevent it falling out of bed, slipping from the waist to the throat. The matter has been satisfactorily set at rest by an inquest on the exhumed body, the medical evidence being so strong as to leave no possible doubt as to the cause of death.

### NURSES FOR INDIA.

On Wednesday last the following ladies sailed for India on duty against the plague: Special nurses—Miss Ellen Smith, Miss J. Snowdon, Miss A. M. Richardson, Miss J. A. Jones, Miss Isabel Grieveson, Miss Blanch Wood, and Miss Lily Foxlee. Eighteen more nurses are wanted, and those who may wish to volunteer will find a copy of the regulations in this number of the "Mirror." The names of the lady doctors appointed are Dr. Margaret Marion Traill Christie, M.D. and B.S.Lond., and Dr. Alice M. Corthorn, M.D.Lond.

### THE NORTH LONDON NURSING ASSOCIATION.

Mr J. McKenzie Davidson's lecture upon the Röntgen Rays at the Highbury Athenæum on the 19th inst. attracted a large audience. Mr. B. L. Cohen, M.P., presided, and Mrs. Cohen also was present. The proceeds were devoted to the funds of the North London Nursing Association. We are glad to note from the report that the committee of the association has been able to purchase and improve the house occupied by the nurses, thus saving in rent £80 a year. The nurses, too, have escaped infectious disease, which is always satisfactory. The work of the association grows rapidly, the staff of six nurses being increased to nine comparatively lately. The doctors of the Great Northern Central Hospital are glad to have the services of the nurses for cases for which, unfortunately, there is not room at the hospital. Over £16 was received in small thank-offerings from grateful patients. The committee draw attention to the lukewarm support of religious congregations.

### THE SURREY NEEDLEWORK GUILD.

The Duchess of York has decided to accept the office of President to the Surrey Needlework Guild, held by the late Duchess of Teck, and a meeting will shortly be held to arrange the work. Lady Ellis, at the request of the Princess, will undertake the active duties in the county.

### THE NURSES' DANCE.

Public opinion is strongly averse to nurses dancing in institutions set apart for the care of the sick—and rightly so to. We are, therefore, glad to see that the minute permitting the nurses' dance to be held in the empty ward underneath the Llewellyn ward, at Swansea Hospital, has been amended, and that the dance is not to take place in the hospital. Doubtless this may prove a disappointment to some nurses who will be unable to attend it if it be held elsewhere, but there must be others who would feel the incongruity of dancing and merriment which might disturb their patients taking place in the hospital itself. We trust that the nurses will enjoy their annual entertainment all the more because it is arranged in such a manner as not to offend the feelings of others on this score.

### QUEEN'S NURSES' ASSOCIATION, DARLINGTON.

The only satisfactory thing about the report of the Queen's Nurses' Association, excepting the nurses' work, which we presume is all that it ought to be, appears to be that the nurses have a home. The debt on the buildings is £700, and the deficiency in the maintenance income reaches £100 a year. This year's Jubilee gifts brought in £313, and the Association has had other help in the shape of the proceeds of a concert and a sale of work. Subscriptions amounted to £292, and the expenses to £425.

### FRAUDULENT MISREPRESENTATION.

The virtue of honesty being the best policy has been pointed out lately by the action of an old nurse named Margaret Rogers to recover £14 7s. 6d. pension alleged to be due to her from the Wolstanton and Burslem Board of Guardians. The poor old lady was verging on sixty, and had held nine appointments under the Poor Law from 1873, and induced the guardians to appoint her as being perhaps a little more than forty-four. When the Superannuation Act passed and she filled up the required form it was found that she was 63, and the guardians requested her to contract out of the Act, and when she refused dismissed her on a month's notice. It might be well to point out that nurses should be very careful to give their correct age on joining the service or it may happen that years afterwards it may be brought up against them that they obtained their appointment by misrepresentation, and are therefore not entitled to a pension.

### QUEEN VICTORIA'S NURSING INSTITUTION, WOLVERHAMPTON.

The Queen Victoria Nursing Association of Wolverhampton, which was founded in 1890, and, of which the new home has been erected in memory of Her Majesty's Diamond Jubilee, has recently been registered to carry on business as described by the title. The management is vested in the Mayor of Wolverhampton and a committee, and there are 200 members each liable for £1. This step has been taken in accordance with legal advice, in order to facilitate the recovery of nurses' fees in case of default. We are sorry to learn that the earnings of the private nurses are employed in maintaining, or in helping to maintain, the district nurses.

### ANOTHER NURSES' ASSOCIATION.

Last summer what is practically a Roman Catholic Nursing Guild, named the "Catholic Nurses' Association," was organised, and the Cardinal Archbishop of Westminster has now formally sanctioned it, and approved its rules. At a meeting held at the Visitation Convent, Harrow-on-the-Hill, a short time ago, 17 candidates received the badge of the Association, and Father Cuthbert gave an address on "Pain." The Association is to provide a religious centre for nurses of the Roman Catholic faith. The rules as to frequency of devotions will not be stringent, nor of such a nature as to withdraw the members from companionship with nurses of other creeds. Members will be able to go into "retreat" at the convent at a moderate charge, and the general meetings of the Association will be held on the first Friday of every month. The badge is a bronze brooch, bearing the title of the Association, "Salus Infirmorum," in the centre.

### A FELLOWSHIP FOR NEWNHAM.

Women who have obtained honours in a Cambridge Tripos examination or the Oxford final school may compete for a fellowship that has lately been presented to Newnham College. It is worth £150 a year, and is tenable for three years.

### THE FACTORY GIRLS' COUNTRY HOLIDAY FUND.

The Lord Mayor has sent a donation of £50 from the surplus of the Princess of Wales's Jubilee Fund to the Factory Girls' Country Holiday Fund. He has promised also to preside at the annual meeting in April.

### NURSES AT KLONDYKE.

Many trained nurses have recently made application to join the forces of several syndicates now fitting out in London for a Klondyke spring expedition. The Chicago Woman's Klondyke Club, shortly about to start for the goldfields, numbers some 20 trained nurses among its ranks.

### THE COVENTRY NURSING ASSOCIATION.

The twelfth annual report of the Coventry Nursing Association indicates a satisfactory state of affairs. In the first place, both the private and district staffs have had more work than they could do. This difficulty in the case of the district nurses was met by the appointment of a fourth nurse. Secondly, the home has been removed from Holyhead Road to the much more convenient premises at 33, Bishop Street; and lastly, money has been forthcoming to pay all expenses, inclusive of those caused by removing and by partly furnishing the new dwelling. A forcible argument urged by the committee for having regular collections at the various places of worship was that it is the opportunity of the poor to contribute their thank-offering for the work of the nurses amongst them.

### SHORT ITEMS.

Miss Maud Danks' concert at St. James's Hall on December 20th in aid of the Benevolent Fund of the Royal British Nurses' Association realised £100. Miss Danks bore the expenses, so that this sum has been handed over to the fund intact.—Nurse Amy Smith of the West Riding Nursing Association has just completed a course of lectures on "How to Nurse the Sick at Home," at the Mechanics' Hall, Stainland, near Halifax. The last one concluded by a competition in bed and poultice making, and the result will be announced at the next meeting, which will be musical. The lectures have been popular.—The maternity Nurse appointed by the Dawlish District Nursing Association at the annual meeting nearly a year ago has been so well received and has had so much work that probably there will be a resolution to establish a second at the forthcoming annual meeting.—Mr. James Gray not very long since presented a nurses' home to the Whitehouse, Whiteabbey, and Greencastle Nursing Association—a most desirable addition. The association is in a flourishing state, and the number of patients during the last year amounted to 259.—The Duchess of Portland has promised to open the nurses' home at Hucknall Torkeard next month. It is a Diamond Jubilee memorial.—The annual meeting of the nurses of the Glasgow Royal Infirmary took place on New Year's Day. Lord Provost Richmond presided, and reviewed the improvements which were in progress, and which would cost when complete £125,000. The meeting was opened by the assembled company singing the One Hundredth Psalm, and was closed by a choir of young ladies singing "O Rest in the Lord" and "In Verdure Clad."

### Lectures to Surgical Nurses.

By H. A. Latimer, M.D. (Dunelm), M.R.C.S. of Eng., L.S.A. of London, Consulting Surgeon, Swansea Hospital; President of the Swansea Medical Society; Lecturer and Examiner of the St. John Ambulance Association, &c.

### XXIII.—DISLOCATIONS—ULCERS.

A greater amount of violence or wrenching aside of a joint than what I have described as resulting in a sprain, causes a dislocation. Here we have a displacement of one or more of the bones of a joint; and the displaced bones occupy some new and faulty position. As a necessary consequence the joint is locked and cannot be moved, and on examining it we find deformity, where the mischief has taken place, and either shortening or lengthening of the limb, as a whole; and pain and swelling will accompany these symptoms. The operation of replacing the bones in their natural position is called "Reduction," and it is one which should never be attempted by nurses. To perform it successfully requires considerable knowledge of anatomy, and although from time to time it is practised with success by uneducated people, such practices are to be discouraged because ill results too frequently follow attempts at reduction made by such folk. All scientific methods for reducing dislocations aim at placing the bones in such a position of freedom that, by the action of the muscles attached to them, they may be brought back into their former situations. Occasionally we are still compelled to use a considerable amount of force over these operations, but as a rule they are performed by manipulation. The surgeons endeavour to realise the steps taken by the bone in its escape from its normal situation, and then by reversing these steps they re-conduct it back to the place from whence it came; such a process is called reduction by "manipulation." Your duties are entirely those of keeping the affected part at rest by bandages or splints loosely applied, until the arrival of the surgeon, and in applying evaporating lotions to reduce swellings and to relieve inflammation which may be set up by the injury; later on you may be directed to rub, or "massage" the joint, so as to restore freedom of movement and to promote absorption of lymph which may have been effused in the damaged part. Having given you my own opinions on the subject of the treatment of sprains and dislocations, I must tell you that hot fomentations are often applied to swollen and painful joints, instead of cold ones, and that they undoubtedly afford much relief and do much good in many instances. To effect this good they must really be very hot, and must be covered with some impermeable material to prevent their cooling by evaporation. Flannel is the usual material employed. A suitable amount of it is steeped in boiling water. It is then taken out of the water and is put in the middle of a towel, which is then wrapped round it to prevent its cooling, whilst all the water is squeezed out of it by twisting the ends of the towel in reverse directions. When this is accomplished the towel is rapidly opened, and the steamingly hot flannel is taken out and is wrapped round the part which it is intended to cover, and then dry flannels or waterproof sheeting are applied over all. The best and quickest way of applying a hot fomentation in my judgment is as follows: A four-folded blanket is arranged on a bed so that its centre lengthwise corresponds with the injured part, when the whole limb, of which the latter forms a part, is laid upon it; when the hot flannel has been wrapped round the joint, one side of the blanket is folded over the limb, and then the other side is brought across and laid over the first layer; by doing this, the whole limb is covered with a thickly-folded blanket, which acts not only in keeping the fomentation hot, but also in restraining movements; it forms, in fact, a blanket splint. To secure it three or four broad pieces of tape encircle it at different points, and are tied by bows. Applied in this way the fomentation is so quickly covered and secured that it has no time to cool, and the heated and moistened flannel which constitutes it will keep hot for some four or five hours. Be careful in one matter when applying these fomentations—be sure that you have squeezed out all superfluous water before putting the flannels on. I have known a nurse scald a patient badly by neglecting to adopt this precaution.

Now let us pass on to a new subject—that of ulceration. The word ulcer is derived from the Latin one ulcus, a sore; and when people tell you that they have sores on the legs or elsewhere they generally mean the same thing that we do when applying the more scientific name to these wounds. Ulcers may occur, and the process of ulceration may go on anywhere in the body, but I shall speak now only of ulcers which affect the skin and mucous membranes, and the tissues beneath them, in parts of the body which you can see. Many causes may be at work to produce them—the action of destructive agents such as heat or cold, or chemical irritants; interruption to the supply of blood or of nerve force; pressure on a weakened surface: or the low form of irritation set up by the presence of a decomposing fluid like urine or offensive perspiration; but the final result is always the same, viz., death of the affected tissue, in small particles. Ulcers have the most diverse appearances, being in some cases ashen grey, purple, bright red, or dirty brown in colour; depressed beneath the level of the part in which they present themselves; on a level with it, or raised above it; having their surface covered with matter, blood, or sloughing membrane. As you meet with the word "slough" from time to time in my lectures, and are told that such and such a part is "sloughing," I had better define what is meant by the term before I proceed further, more especially as the process known by this name is the essential one in ulceration. A slough is that portion of dead matter which is separated from sound flesh; and when any tissue of the body is described as "sloughing," the meaning of the expression is, that that tissue is dying and is being separated from the living flesh which surrounds it. Strictly speaking, when you see the dark brown or grey mass which characterises a slough, you are looking at a piece of mortified flesh, and such a condition of affairs marks gangrene; but the difference between gangrene and ulceration is only one of degree, death taking place in a mass of flesh in the first case, and in little bits in the second; but, all the same, if ulceration is proceeding very rapidly, you will often see sloughs on the surface of the wound.

The amount of pain accompanying ulceration is very variable. In some cases it is very great, and then it is due to the sensitive ending of a nerve being involved in the wound. When mucous membranes are affected in this way, and especially when those lining the end of the bowel, the edges of the tongue, and the corners of the mouth are concerned, the suffering caused is often very intense. This results from two causes—the fact that these parts of the body are continually being moved, and that discharges of irritating quality are flowing over them. The constant movements to which ulcers at the anus are subjected by the action of the round muscle underlying them, prevents their healing, and, in order to cure them, surgeons are often compelled to make a cut across them, of a sufficient depth to divide the muscle, and so set it at rest. As far as you are concerned, your interest is not so much in the variety of ulcer which you see, as in its condition. You should know whether it is being healed, or not, or if it is extending. A healing ulcer has a healthy-looking red surface made up of small red granulations, and the discharge from it consists of that sweet-smelling thick matter which your experience in the wards of the hospital tells you is formed from the blood when wounds are uniting by "second intention." Its surface is level with the surrounding skin, and this skin is natural in appearance, and is free from any inflammatory redness denoting congestion of the blood-vessels; moreover, a bluish-white pellucid margin is present at its edges, intervening between the skin and the raw flesh.

### Diet for Consumptive Patients.

GENERAL PRINCIPLES.

FAT and oil are essential articles in the diet for patients suffering from phthisis, but how to render them palatable is often a difficulty. Patients should be induced to take as much as possible; therefore their meals must be most carefully prepared, all should be of a nourishing nature and such as can be easily digested.

Brown bread and butter will be found useful, varying the kind of bread every few days. Be very careful that the butter is of the best. Cream, which ranks next to cod liver oil in utility, can be given in a variety of ways. A little red currant jelly added to a wineglassful of cream early in the morning is often liked, when cream alone would be distasteful. Bread lightly fried in the hot fat from bacon is another excellent method of supplying fatty foods. It is sometimes necessary to malt farinaceous foods. This is easily done by using the following recipe, which I give in full from Mrs. E. Hart's book: Mix three ounces of crushed malt thoroughly well with half a pint of cold water in a jug. Let the mixture stand over night. The supernatant liquid is then carefully strained through two or three folds of muslin, until it comes through fairly clear and bright. Malt infusion thus prepared has a light brown colour like sherry, a faint maltish taste, and the odour of beer-wort. It is prone to fermentation, and should be made fresh every day. One tablespoonful will digest half a pint of gruel.

RECIPES.

BAKED TROUT.—Scrape the scales off a fresh trout and cut off the fins; take out the entrails and well wash the fish in salt and water. Wipe it dry with a clean cloth, and make some small slits in the skin; rub it over with salad oil and sprinkle it with pepper and salt. Place the fish on a tin and cover it with a well-buttered paper, bake it for about 15 minutes, take up, and dish on a very hot dish. Sprinkle with chopped parsley, and serve lemon or any piquant sauce with it.

MUTTON CUTLETS.—Trim the best end of a neck of mutton and cut from it some neat cutlets; lard them with small lardons of fat bacon, steep the cutlets in salad oil, then grill them on the unlarded side for three minutes. Put the grill on a baking tin and put them just as they are in a quick oven for five minutes; take them up, and dish on a border of mashed potatoes, pouring some tomato sauce round the base. Serve as hot as possible.

FRIED OYSTERS.—Take some large oysters, beard them, and put them on a plate and sprinkle them with pepper and salt, a little lemon juice, and pour over them a little warm butter. When the butter is cold dip the oysters separately into flour, then into beaten-up egg, and lastly, put them into rolled biscuit crumbs (crackers are the best); smooth them with a knife, then fry in hot butter till a pale golden colour; serve quarters of lemon with the oysters.

STEWED TRIPE.—This is a most nourishing dish. If tripe is properly prepared few dislike it; great care must be taken in cleansing it properly, blanching and cooking it very slowly for ten hours. It is then ready for making into various dishes; the following is one: Cut up a pound of cooked tripe into square pieces, put it in a stewpan with half an ounce of butter, one onion, a little bunch of herbs, two ounces of bacon cut into dice shape, and two fresh mushrooms. Fry altogether till a light brown colour, then shake in a tablespoonful of flour, a quarter of a pint of white wine, half a pint of water, and a good teaspoonful of Bovril. Cook very slowly for an hour and three-quarters, remove what fat is on the surface, dish up as hot as possible.

DEVILLED BRAINS.—Calves' brains plainly served are rather insipid, and though so good for them, patients soon tire of them. Cook them in this way and they will be thought delicious. Cut a small round of bread, toast and butter it, and then sprinkle a little grated Gruyère cheese on it. Cut a slice of cooked calves' brains and lay on the toast. Spread this very thinly with a little English and French mustard. Sprinkle a little more cheese on it, and some browned bread crumbs. Put all in the oven to get quite hot and the cheese melted. Be careful not to over-cook it, as it will only take a minute or two. Serve on a dish paper and garnish with watercress.

TENDONS IN WHITE SAUCE.—Well wash a calves' foot and tie it in a cloth, put it in a large saucepan with some cut up vegetables and cover with cold water. Bring it quickly to the boil, skim, and simmer gently for four hours or till the tendons are tender. When cooked, take up, split open the foot, cut the tendons into little square pieces, sprinkle them with chopped parsley, and mix sufficient hot white sauce with them to moisten. Put them into china cases, and garnish with little rings of fried bread.

CHOCOLATE CREAM.—Put two ounces of finely cut up vanilla chocolate into a stewpan with a quarter of a pint of hot water and one ounce of icing sugar. Bring to the boil, then stir in a quarter of an ounce of cornflour that has been mixed smooth with a little cold water. Stir till it boils, then strain into a basin, and when cold add a quarter of a pint of stiffly-whipped cream that is sweetened and flavoured with a little vanilla essence. Mix lightly, and serve in custard glasses.

A SOLID CUSTARD.—Put into a stewpan half a pint of milk, two ounces of castor sugar, and bring to the boil, then add a small quarter of an ounce of leaf gelatine (Mrs. A. B. Marshall's is the best); when dissolved stir it on to three yolks of eggs that are mixed in a basin. Then return the mixture to the stewpan, and stir over the fire till it thickens, but on no account let it boil. Strain it, and when cool, but not set, stir in a quarter of a pint of whipped cream that is sweetened and flavoured with maraschino or any flavouring that the patient likes. Put into a fancy mould, and when the custard is set dip the mould in warm water and turn out on to a glass dish.

Amongst vegetables for consumptive patients asparagus, peas, French beans are considered some of the best; but as these are not in season now we must take what we have. Young Brussels sprouts are very good; also stewed celery and winter spinach. All these help in making a healthy diet, and do their part to build up the wasted system. Boiled potatoes are dull day after day; if fried occasionally in ribbon form or Saratoga chips they are a change, and are always liked.

As to strength-giving drinks, milk, of course, comes first, either plain or with soda-water in it. Koumiss is good when ordinary milk will not digest, or it can be malted to make it so. Cooling drinks have been given in a former article.

### Mrs. Scharlieb at the Grosvenor Crescent Club.

ON January 25th Mrs. Scharlieb, M.D., gave an interesting address to the members and guests of the Grosvenor Crescent Club, in connection with the Women's Institute, on "The History and Growth of the Medical Movement Amongst Women." Mrs. Bamford Slack took the chair. Mrs. Hays Hammond proposed the vote of thanks, and Mrs. Garrett Anderson, M.D., in seconding it gave some valuable hints to intendant students of medicine. Dr. Baume, of New Zealand, made a few humorous remarks upon the attitude of men towards the wider ambitions of advanced women.

### The Workhouse Probationer.

The workhouse probationer is face to face with at least two grave dangers. The one, the moral risk of moral deterioration from her contact with paupers; the other, the impossibility of obtaining a certificate from any of the numerous workhouses which have not the proper qualifications for training.

At the last meeting of the Workhouse Infirmary Nursing Association Miss Catherine Wood urged as one of the reasons why that body should continue its work that in consequence of the Local Government Board's order to abolish the pauper nurse the guardians would replace her by young women of from eighteen to twenty years of age.

However strong may be our sympathy with the sick and infirm of our workhouses, however desirous we may be of bringing into their lives all that science and human tenderness can give, we must never, in justice to the young and strong who have yet to play their part in the world, forget that paupers are often, for one cause or another, the failures of their generation. They are failures generally because of incompetence, self-indulgence, and vice—for careful inquiries into the root of extreme poverty have resulted in bringing to light overwhelming evidence that comparatively few worthy respectable people reach these refuges for the destitute.

The decade between fifteen and twenty-five years is perhaps the most important in life. Then, as a rule, the child has shaken off the restraints of childhood and become a positive factor in society at large. The latent possibilities for good and evil only need the stimulus of environment to develop and become definite characteristics of the individual; habits, too, are formed that are rarely broken. These are hard facts, and cannot be disregarded by those who would be just to all. Some might even maintain that it would be better that those that are paupers should shuffle through the dregs of a wasted life entrusted only to the mercies of their kind than that they should contaminate new young and healthy minds, which in their turn may become the wasters and not the makers of their day. But such an alternative is not the only one before us. There are other things that may be done. It is not impossible to obtain the help of older women to whom the objection just urged would not apply True, it may cost more money, but it works out cheaper in the end.

The second danger in the path of the workhouse probationer is that all workhouses have not the facilities to train nurses, and if she be not careful to select one that has the power to confer a certificate she finds herself at the end of her novitiate in exactly the same position as she was when she started as far as chances of promotion are concerned.

To would-be nurses, therefore, we would advise that they should occupy their earlier years acquiring the housewifely arts of cooking, needlework, and housekeeping, all of which will serve them in good stead later, whatever walk in life they enter on. They could also with advantage, if they have the means and opportunity, improve their minds by a liberal course of reading, and study some accomplishment for their own recreation. A knowledge of some foreign languages is certainly a factor in promotion in the army and navy hospitals.

When the age approved by the best training schools is reached, well brought-up candidates have no difficulty in obtaining admission, and the only care ought then to be in the judicious choice of the school best suited to individual requirements.

The objection that will probably be raised against this advice is that many candidates cannot afford to wait so long. This is a fallacy. There is nothing so expensive as to work without the prospect of progress in one's profession and one's career. A good, strong girl leaving school can command quite as high wages as a kitchen-maid, and will receive excellent preparation in that position for a most important branch of nursing; or, as under nurse in a good family, she will have opportunities of learning how to deal with children; or, as under ladies' maid, she can acquire dexterity with her needle and at the toilet. Young ladies who need not become breadwinners at so young an age can study pharmacy, dispensing, cookery, with subjects of more general interest, and thus guard themselves against one of the greatest drawbacks to a nurse's life from a social point of view. Indeed, the more one thinks of it, the more certain does it become that there is no accomplishment nor womanly learning that comes amiss in the comprehensive art of nursing.

### Appointments.

### MATRONS.

Albany General Hospital, Grahamstown, South Africa.—Miss Mary Leigh Swift has been appointed Matron of the above hospital. She received her training at Guy's Hospital, London, from 1889 to 1892, and since then has held the posts of charge nurse at the Fever Hospital, Grafton Street, and the Mill Road Infirmary, Everton, Liverpool, and the Northern Fever Hospital, Winchmore Hill, London, N., where she was appointed night superintendent of nurses in February, 1896, which position she fulfilled until December, 1897.

The Workhouse, Gravelly Hill, Birmingham.—On January 18th, Miss Katherine Platt was elected Superintendent Nurse of this workhouse. She was trained at the Edinburgh Royal Infirmary, and has previously held the following posts: Superintendent nurse of the Hull Workhouse hospital, nurse at the Oldham Infirmary, and district nurse Blakewell, Derbyshire.

Cumberland Infirmary, Carlisle.—Miss Wemyss, who was appointed Matron of the above infirmary on December 16th, has been compelled to resign before having taken up her duties there on account of illness. Miss Fawcett was therefore appointed Matron on the 19th inst. She was trained at the London Hospital, and is now home sister at the Poplar Hospital for Accidents.

Chalmer's Hospital, Edinburgh.—Miss Mary D. Stephenson has been appointed Matron of the above hospital. She was trained, and for five years sister, at Charing Cross Hospital, and has been matron of the Tiverton Infirmary for the last fourteen months.

East Grinstead Cottage Hospital.—Miss Agnes G. Morton, who was trained at Guy's Hospital, and who has lately been matron of Axminster Cottage Hospital, was appointed Matron of this hospital on January 18th.

### Minor Appointments.

Hospital for Women and Children, Leeds.—Miss Rebekah E. Wheeler was appointed on January 1st Sister of this institution. She was trained at the General Hospital, Wolverhampton. She has been engaged in private work, as charge nurse of the women's wards in the Clinical Hospital, Manchester, and also at the District Hospital, Walsall.

St. Giles' Infirmary, Camberwell.—Miss Louise Alexandra Borĕt has been appointed alternate night and day Nurse of this infirmary. She was trained at the Consumption Hospital, Brompton, and has been ward nurse at St. Saviour's Union Infirmary, charge nurse Croydon Infirmary, and nurse at Hackney Infirmary.

Chelsea Hospital for Women.—On January 29th Miss May Dods became Charge Nurse of this hospital. She has a three-year certificate from the Infirmary, Lewisham.

### Nursing the Plague.

### MOVING CASES TO A FRESH CAMP.

By a Trained Nurse.

We have not been in want of excitement at Poona; we received notice that a site for a new camp had been fixed, and that we were to move into it as soon as it was finished. It was a great source of anxiety, more especially as at first only the convalescents were to be removed, and all new cases were to be taken to the new camp, while my head-quarters were at the old one, I going to and fro three or four times a day as needed. The move of the convalescents was safely accomplished in bullock carts, their attendants following on foot, the whole line of march looking most comical. I drove on in front, and saw them settled in their new chuppers. Then came the rub; if the fresh attacks were to go to the new camp they certainly needed looking after, so when two most unsatisfactory days had passed permission was given to move the invalids up also. No one seemed the worse for it, though several do not ever recollect having been moved at all. The chuppers (of grass and bamboo) are much nicer than our old ones, and are built in lines. There are eight of them.

Our observation cases tents have some strange occupants at times; of course, in an epidemic like this many cases are brought in needing careful nursing, but having little in common with the prevailing disease. For instance, we have at present a case of paraplegia. He was seen to fall apparently without any reason, so notice was sent to the search party, who promptly brought him in to us. He needs carefully looking after of course, but still does not count as a patient. Other observation cases have been jaundice, ague, erysipelas, &c., most of them recovering in spite of their surroundings. My patients are all very happy, singing, playing cards when they are able to sit up, or telling long tales, all beginning with the invariable "Once upon a time." I cannot imagine how the nurses who come from home, knowing no Indian language, manage. I, who speak both Hindustani and Mahratti, have always been able to gain my point with conscious patients. We are great friends; if I obtain leave for two hours I am greeted with quite an ovation on my return.

Two cases of plague occurred, both proving fatal, in which inquiry led to the fact being revealed that the father had in each come to the house where the children were from a plague-infected district.

One of the cases—a little girl of seven—was admitted with a temperature of 103°, and an almost imperceptible swelling in the right axilla. She was quite unconscious, and the pulse could hardly be felt. Ice to the head and the usual medicines were prescribed. Two days passed, during which the temperature ranged from 104° to 100·2°; the child never regained consciousness, was extremely restless, and was fed with the greatest difficulty.

On the third morning, about ten o'clock, the patient was bathed in a profuse perspiration, the eyes were wide open, the whole of the right side of the chest and neck became swollen, there was great difficulty of respiration, and the pulse was quite imperceptible. From the first the child could not bear the gentlest examination of the gland without shrinking. Bowels were very constipated, vomiting very persistent. A child who lived in a hut between the dwellings of the above-mentioned children has now developed the disease.

The fathers of the two fatal cases (and apparently the conveyers of the fell disease) are in perfect health.

### Animals Attacked by Plague.

A stray dog had attached himself to the hospital, and when the camp moved turned up at our new home. He seemed very listless for a few days, and then I noticed that he experienced difficulty in swallowing. On examination I found a gland on the right side of the throat. It burst in three days' time, but on the fifth day the animal died. The swelling did not interfere at all with his bark, only the poor creature became a perfect skeleton. The dog had lived day and night for three months in the plague camp before he fell a victim to the disease.

### TERMS OF ENGAGEMENT.

The following are the terms of engagement which are issued to candidates by the India Office:—

1. Selected candidates to be termed "lady nurses."
2. Candidates to be between 28 and 35 years of age, and to produce certificates of birth.
3. To be examined by the President of the Medical Board (before engagement) as to fitness for service in India.
4. To produce certificates of at least three years' training at a hospital in which adult males receive medical and surgical treatment, and in which a nursing staff is maintained. Also to furnish recommendation from the matron of the hospital at which they have been trained.
5. Engagement to be for not less than six months, except in case of misconduct or breakdown of health. Notice of one month to be given before termination of engagement on the part of Government or the lady nurse.
6. First-class railway ticket and free passage by second saloon P. and O. Company's steamers to India and back; but resignation within a year, except on account of ill-health, not to entitle to a free return passage from India.
7. Pay: Rs. 175 per mensem, in addition to free quarters, fuel, lights, and punkah pullers; to commence from date of embarkation to India.
8. Outfit allowance of £10.
9. Travelling expenses from and to residence in England to port of embarkation (claims for conveyance of baggage to be supported by vouchers); 6 cwt. of baggage allowed.
10. To embark for India within one week of appointment.

Applications to be made to the Secretary, in the Revenue Department, India Office, Whitehall, S.W.

### Our American Letter.

A point of some importance is at present receiving attention in the American nursing world. It has arisen out of the refusal of the Board of Governors of the Orange Training School of Nurses to give Miss Niles, one of the nurses trained at that school, her diploma, and she is suing them for it. Some of her adherents argue that governors have no legal right to withhold her diploma if she has passed the required examinations, and is in point of training an accomplished nurse. Others take a more sensible view and think that guardians are right to withhold the diploma if the woman fail in moral qualities that are equally as important as technical skill. Granting this, there still remains the ticklish point of deciding what moral qualities are requisite and what are not, and governors have a wide field whereon to draw the line. We in London know something of the constructions that can be put on "unseemly conduct," consequently a legal definition would materially help the cause of justice. The reasons given for their refusal in the case of Miss Niles are reported to be trivial, and if this be so she deserves to come out of the ordeal with all the sympathy possible, for she will have done yeoman service in the cause of nursing. If, on the other hand, the governors show that they have reasonable grounds for their refusal, though Miss Niles will suffer, it will be a warning to other nurses not to be too confident in training alone to ensure their after success, and will certainly add to the value of a certificate.

### A Book and its Story.

### "THE GADFLY."

To say that novelists show less originality than any other class of writers may sound like a paradox. But anyone who not only reads novels for the purpose of filling up a few vacant hours but who studies them as a branch of literature, must have observed that there is a fashion in novels, just as there is in the cut of dresses and the shape of hats. Three or four years ago foreign influences were at work. Zola and Daudet set the fashion for English novelists, just as Worth set the fashion for English dressmakers; and for a time it seemed as if the pure and invigorating stream of English Romance was about to lose itself in the fœtid and poison-laden quagmires of French Realism. Suddenly there came a change. In literature, as in the affairs of politics and commerce, the interests of Englishmen are essentially varied. Their robust common sense revolted against a view which implied that one topic alone occupied the minds and influenced the doings of men and women. The sickly perfumes were dissolved in air. There was a demand for something healthier. The novel of adventure, of war, of political intrigue, of romance, in the old-fashioned sense of the word, began to appear, and hence it has come to pass that so large a space is now occupied by the works of Stevenson, Conan Doyle, Stanley Weyman, Crockett, and their host of imitators.

The volume before us* is one of many historical novels which have been recently published; and the author is to be congratulated on his choice of a subject and the manner in which he has treated it. The story begins somewhere about the year 1833. Arthur Burton, an English lad of 18, is studying in a theological seminary at Pisa, of which Canon Montanelli is the Father Director. "He was a slender little creature, more like an Italian in a sixteenth century portrait than a middle-class English lad of the thirties. From the long eyebrows and sensitive mouth to the small hands and feet, everything about him was too much chiselled, over-delicate. Sitting still, he might have been taken for a very young girl masquerading in male attire; but when he moved his lithe agility suggested a tame panther without claws."

An orphan, under the charge of Mr. James Burton, his step-brother and his wife, who are strict Protestants, while he is a Catholic, the young fellow's education has been entrusted to Montanelli, to whom he is devotedly attached. He has, however, joined one of the secret societies which had been formed for the purpose of delivering Italy from the Austrians, along with one of his friends, Giovanni Balla, and a young girl, Jennifer Warren, whose Italian schoolmates call her by the pet name of "Gemma." Montanelli is appointed a Bishop, and goes to Rome. A new director, Father Cardi, takes his place; and to the man Arthur, under the Seal of the Confessional, admits that he is a member of the Young Italy Society. Soon after this he is arrested, but though treated in the harshest manner, he refuses to betray his associates. At last he is released, but by a misunderstanding Gemma Warren is led to think that he has betrayed their friends. She meets him on his way home, and though she loves him, wrenches her hand away from his, and strikes him across the cheek.

He goes to the Burton's house, where he finds his relations so angry with him that a family scandal is suddenly and cruelly revealed. "It's quite time," Mrs. Burton exclaimed, "he did know what his mother was; why should we be saddled with the child of a Popish priest? . . . There, then, look!" She pulled a crumpled sheet out of her pocket and tossed it across the table to Arthur. He opened it; the writing was in his mother's hand, and was dated four months before his birth. It was a confession addressed to her husband, and with two signatures; one was his mother's, the other was Montanelli's. The young man seized a hammer, breaks to pieces the crucifix before which he had often prayed. He writes to Montanelli—"I believed in you as I believed in God . . . and you have fooled me with a lie." Then he goes away with a laugh and a shrug of his shoulders as he passed his mother's portrait. She, too, had lied to him. That evening he disappears, his hat is found floating in a canal, but no other trace is found of him.

Thirteen years pass; and the thread of the story is taken up again in 1846, soon after Pio Nono has become Pope, and when the whole of Italy was seething with conspiracies against the yoke of Austria. In Florence the arrival is expected of one Felice Rivarez, who is coming from South America to help them. He is known as the Gadfly, of whom the police have published a description, part of which read, "Special marks, right foot lame, left arm twisted, two fingers missing on left hand; recent sabre-cut across face; stammers." He is, we are told, a journalist, a satirist, who is coming to Italy to support the revolutionary movement, and in particular to wage war against the clergy. In due time the man arrives, and is welcomed by the conspirators, among whom is Gemma, now the widow of Giovanni Balla. "He was swarthy as a mulatto, and, nothwithstanding his lameness, as agile as a cat." Maimed, mysterious, a cripple, and scarred with wounds, this mysterious Gadfly turns out to be Arthur Burton, who had fled to South America, and, after suffering every hardship, now returns to Europe. At first not even Gemma recognises him; but it gradually dawns upon her who he is. Two days after his flight she had found out that she had been wrong in accusing him of treachery, and the idea that she had driven him to his death had haunted her since. When she discovers that he is still alive, she does all she can to make amends. The secret also comes to the knowledge of Montanelli, who is now Cardinal Montanelli; and the main interest of the plot, apart from the intrigues of the Young Italy party, centres round these two—the father and the son—the father, whose life had been darkened by his shameful past, and the son, whose soul is lacerated by the memory of that old betrayal, which had destroyed his faith in human truth and goodness.

This is a genuine romance—amongst the best which has been published lately. It is filled also with a deep pathos, of which the following is an example. "The Gadfly" is in prison, and Montanelli offers to help him to escape, and then to kill himself. "Will that content you?" he asks; "it is all I can do. It is a great sin, but I think He will forgive me. He is more merciful——." The Gadfly flung out both hands with a sharp cry, "Oh, it is too much! What have I done to you that you should think of me in that way? As if I wanted to be revenged on you! Can't you see that I only want to save you? Will you never understand that I love you?" He caught hold of Montanelli's hands and covered them with burning tears and kisses. "Padre, come away with us? What have you to do with this dead world of priests and idols? Padre, I have always loved you—always, even when you killed me; will you kill me again?" Montanelli tore his hands away. "Oh, God, have mercy on me?" he cried; "You have your mother's eyes!" A strange silence, long and deep and sudden, fell upon them both. In the gray twilight they looked at each other, and their hearts stood still with fear.

* "The Gadfly." E. L. Voynich. (Heinemann. 1897. London.)

### Everybody's Opinion.

[Correspondence on all subjects is invited, but we cannot in any way be responsible for the opinions expressed by our correspondents. No communication can be entertained if the name and address of the correspondent is not given, as a guarantee of good faith but not necessarily for publication, or unless one side of the paper only is written on.]

### TYPHOID FEVER.

"M. S." writes: A nurse who fully appreciates the papers on Typhoid Fever in The Hospital, by "A Trained Nurse," would be glad if the writer could give her any information as to how she managed about leaving her patient when doing her sick cookery; also whether the relatives or friends have any objection to her being in the kitchen, as there is usually great fear of infection.

### A PATIENT'S VERDICT.

A grateful patient writes of the nurses and life at Charing Cross Hospital, where he had been nursed through typhoid fever. "All I can say of hospital nurses from my experience is that a more patient, Christian like, nice, lady-like lot of girls do not exist. As for the life in hospital, some would perhaps, find it rather rough. There are all sorts and conditions of men there, and the idea of drinking tea out of enamelled mugs and eating thick bread and butter when convalescent would not suit some; but all I can say is, that if I should be unfortunate enough to be ill again I should ask to be taken to hospital."

### A DISTRICT NURSE'S PAY.

"Policy 443" writes: I have read with deep interest the letter in The Hospital with regard to salaries for district nurses. I was a district nurse, and my salary was £65 a year. I paid 8s. in summer for two rooms, with attendance, and 10s. in winter. My food cost me 7s. or 8s a week; tithes, 2s. 6d.; laundry, one week with another, 2s.; Pension Fund, 1s. 2d.; papers, 4d. Out of the other 3s. 6d. per week had to come uniform (and boots are a very serious item to district nurses), in fact all clothing, and holidays, so I would very much like to hear how 10s. per week could be saved out of it. My rooms and food were comfortable, but no luxuries in any way could I afford. I think the subject very interesting indeed, and if one and another bring forward their experiences may be very helpful to one another. I may add in conclusion that I would rather lose a glass of milk than miss my Hospital Friday morning.

F. Taylor writes: Will you allow me to say to "E. C." through your paper how sorry I am that she should have thought I meant anything unkind. Nothing could have been further from my wishes. I quite agree with her that economy is one of the dreariest of the virtues. It is only the fear of a worse state of things that makes me practise it. I think it noble of her to have supported anyone else. I guess from her wail, "one monotonous round of work," that she's a "young 'un," and I sympathise heartily with her, being a very old stager myself, one of Lady Augusta Stanley's first twelve in the Trained School for Nurses started in 1876. To my mind a woman's first duty is to try and get married; there is a far better chance as waitress, say, in a ham and beef shop, and it's a much more cheerful life. May I say to her, why does not she bank her savings, and then they are always hers! It is only poor weak man who cannot save unless he is banded together. If she has a returnable pension she only gets 2½ per cent. on her money, whereas she could get that while it was being gathered together, and then when she had enough she could invest it and get 4 per cent. safely with a decent investment. If she has gone in for a cheap pension she loses it all if she dies beforehand; in that case she will only have been working for the Pension Fund, and if she only lives to draw a quarter's annuity all the savings of years also vanish, she loses the pleasure of leaving them to help someone else whom she knows. Much as I respect the Editor of The Hospital, and thank him for giving us such papers as Mr. Stanmore Bishop's on "Operations in Private Houses," I am afraid I can't disclose all the little secrets of my economy to him, and have often been neither good-natured nor unselfish in exercising it I fear.

*** We are sorry F. Taylor cannot disclose the secrets of her "economies," as they would have perhaps been helpful. Whether it is a "woman's first duty to try and get married," is purely a matter of opinion. With regard to her remarks on the Pension Fund, she seems to overlook all the advantages accruing from bonuses and the Junius Morgan Benevolent Fund, also the fact that she can will her savings to whomsover she wishes should she not want them herself, can withdraw principal and simple interest at 2½ per cent., at whatever age she likes, and may invest them as she chooses. Therefore to stand alone is not cheapest, even from her own point of view. Lastly, with regard to the monotonous grind of nursing, we would ask her to remember "that whosover giveth but a cup of cold water in the Master's name shall in no wise lose his reward." It is the spirit of serving the Lord "who givest all" that elevates the nurse into a ministering angel, and to such the reward is sure.

"Barnet" is thanked for her friendly note. She will see by last week's "Everybody's Opinion" that we were right.—Ed. *T.H.*

### SOCIAL PRIVILEGES OF THE WORKHOUSE NURSE.

"A Mental Attendant" writes: Considering the valuable services that have been rendered to, and the sympathetic interest that you have always taken in the cause of anything appertaining to nurses, I venture to solicit a little space for the correction of a statement which appeared in your issue of the 8th inst., and which in my opinion is calculated to create a false impression in the minds of your numerous readers, apart from the harmful effect it is likely to have upon those immediately concerned. The paragraph I refer to is that concerning the officials of the Kingston Union. The nurses have one large recreation-room, which is situated on the male side of the house (not in the infirmary), and to this room those nurses who are off duty adjourn between the hours of eight p.m. and ten p.m. for a couple of hours' recreation. Occasionally the male officers, two in number (at the invitation of the nurses and with the consent of the master), have been invited to take part in an impromptu concert. Now, considering that the duties of these officers brings them into daily contact with the nurses, what exception can any reasonable minded person take to a couple of hours spent in trying to forget the worries incidental to every Poor Law official, who are called upon at all hours day and night to perform many unpleasant duties of which the outside public are entirely ignorant, for which they receive in many cases a bare pittance, and are expected to maintain a respectable appearance. Some gentlemen, it would appear, seek to make the workhouse an even less attractive place for their officers than it rightly should be for the pauper inmates. However, thanks to our late chaplain, the door of the nurses' recreation-room is closed, and the male officers will have to derive whatever comfort they can from a tête-a tête in their mess-room, which is not large enough to swing around the proverbial cat, and which is devoid of anything in the way of comfort or recreation.

### THE ROYAL BRITISH NURSES' ASSOCIATION.

Dr. Hugh Woods writes: Dr. Bezly Thorne says that I have not answered him. He forgets that one of the well-established methods of answering a certain class of questions, a method usually associated with the name of Socrates, a method which was sanctioned by the example of the founder of Christianity, is to answer one question by another. I quite appreciate the difficulty he feels in answering the question, Why did the Royal British Nurses' Association give an *ex-officio* seat on the Executive Committee to the President of the Incorporated Medical Practitioners' Association? Dr. Alderson says he is able to confirm Dr. Thorne's statement that no promise was made, because, if so, why did he not hear of it? This is like defending a man accused of a crime by bringing up witnesses who did not see him do it. Dr. Alderson, however, answers the question which Dr. Bezly Thorne fears. He says the seat on the

Executive Committee was conferred "as a graceful compliment to the general practitioners and to the representative character of their society." Dr. Alderson, having given this explanation, naturally feels it necessary to show why the attempt has since been made to reverse this "graceful compliment." He then proceeds to say, or insinuate, that it arose from my action in publishing, on behalf of the Association of which I have the honour to be president, a certain well-known document. Dr. Alderson overlooks the fact that the proposed alterations in the constitution of the Royal British Nurses' Association had been drafted by the officials of that Association before anyone knew that I was likely to be president of the Incorporated Medical Practitioners' Association. Dr. Alderson, a little time back, asserted that I had acted without the sanction of my Council, and on my proving the reverse, and taking a vote of the Council in regard to the matter, he thought it necessary to resign. The fact is that Dr. Alderson, though an excellent man, has a way of his own of mixing past and present, cause and effect, fact and fiction, into a homogeneous emulsion remarkable for its opacity. But to return to Dr. Bezly Thorne. He says it is "beyond the power of man" to substantiate the allegation I have made. I am but a man, and therefore I despair of substantiating my allegation to his satisfaction. I have inquired into the matter, as I promised, and I have done what I could to meet Dr. Bezly Thorne's objections, but as he gives the lie to me and Dr. Bedford Fenwick, and contents himself with descanting on the courage with which he himself speaks the truth, it seems to me that we have passed from the region of reasoning. As to me "not daring to take up the glove that has been thrown before me," I am not a pugilist, and I would not wish to hurt a hair of Dr. Thorne's head. I much prefer to watch his cherub face as he "pricks rhetorical bubbles with the point of truth." I never accused the officials of the Nurses' Association of "treachery." I accuse them of conduct not uncommon on the part of candidates at elections—I mean making promises with a view to obtaining support, and then conveniently forgetting them when they have served their purpose. In conclusion, let me say how sad it is to see Dr. Bezly Thorne falling out with the "officers of the nurses' choice" at the last Council meeting, and leading a small, if not turbulent, minority. He is dissatisfied with the management of the Royal British Nurses' Association, and so am I. Under the circumstances, instead of putting on the glove thrown before me, I take off my metaphorical glove to shake metaphorical hands with Dr. Bezly Thorne on the occasion of his secession to the "minority."

*** A desire to be fair to both sides leads us to publish the above letter. We cannot, however, refrain from saying that the chief interest of our readers in the correspondence is likely to centre on the point which is left out. Dr. Hugh Woods made a certain statement in regard to a compact or arrangement made between the representatives of the Royal British Nurses' Association and the Incorporated Medical Practitioners' Association. Dr. Bezly Thorne denies that such a bargain was ever made, and challenges Dr. Hugh Woods to substantiate his statement. Instead of doing this Dr. Hugh Woods repeats his assertion, and asks a question. Clearly a correspondence cannot be allowed to continue on such a basis. If Dr. Hugh Woods can answer Dr. Bezly Thorne's challenge we shall be glad to give him the use of our columns, but if not there would seem nothing more to say, and we think this correspondence had better cease.—Ed. *T. H.*

### THE TRIALS OF THE MIDDLE AGED.

Nurse Thompson writes: You have always so kindly helped the nurses that I venture to ask your advice. I have turned 47 years of age, and I am thinking people find I am too old for nursing and, moreover, have not long left South America. Another thing is that the lady here sees that the midwifery work did not cover my expenses. I would have kept it on myself if I could have made it pay. Unfortunately the doctor does not like trained nurses; I have a very nice testimonial from him, and he speaks well of me, so I am told; but I think he believes he would get my cases and there would be more money for his pocket. I am sure if he knew district nurses that he would find he would have more money and more comfort in their help. Is there any occupation suitable for me? One of your papers speak of mothering. What is sanitary inspector's work? I know it is understanding drains and ventilation and visiting houses, but it seems to me to be a prying business and would want a lot of tact to manage the people and also money and brains to pass the examinations; and massage, I suppose, would also want money, &c. I am too old for that, but perhaps I might learn it to teach others; and I am, I suppose, too old for Zenana work. I should like to give a word of caution to nurses abroad wishing to take up nursing in England—"Don't." It is a short word, but very expressive. People look upon you as strangers and foreigners when you go to them for work. It is the same, and worse, than taking up work in a foreign country. When I arrived in London after eight years' absence I was surprised to see so many nurses in uniform flitting about like bees in a hive. Then I began to realize that I did wrong in coming to England. It seems to me that, like frogs, the young kill the old ones. Not that I mean to disparage the nurses, for I have always found them very kind; I might put it in another way—the survival of the fittest. Another thing that is much altered are the unions. There is difficulty in obtaining nursing or matronship even in them. I have taken great interest in the nurses' housekeeping, &c. It is kind and charitable to help one another. A nurse may know how to do the thing required, but the cleverest doctors and nurses are always willing to enlarge their knowledge. I think the author of "Thrift" a little hard; does she not know human nature is prone to hide itself sometimes, and those that are seemingly careless are the reverse. There are few nurses that have not some burden to carry and someone to help out of their slender purse, and none except the One above knows of it. Nurses I am sure would be better pleased to have their housekeeping done privately, arranged by letter like Miss Mena Bielby had such forethought in acting upon, for I am quite sure the discussion has done more harm than good. I find £30 and furnished rooms are offered now. I call that sweating. We want another Charles Kingsley or a Dickens to write for us. Or if there could be a union such as the Trades Union to fix and arrange salary, &c.

*** We sympathise with Nurse Thompson. She has to face all the trials common to her calling at her age without the advantages of having built up her connection. She has not given us any information as regards her training, so that it is impossible to give her advice. One thing seems plain, however; she is an experienced midwife, and, as such, she ought to be able to earn her living either in connection with district or institutional work or privately. And she would certainly find as many or more difficulties in any other branch of the profession, plus the difficulty of learning, which is considerable at her age. In the advertisements in the "Mirror" for January 22nd there are five, if not six, advertisements that might suit Nurse Thompson.—Ed. *T. H.*

### Where to Go.

The second sessional lecture of the Royal British Nurses' Association will be delivered at 11, Chandos Street, Cavendish Square, W., on Friday, January 28th, at eight p.m., by Dr. Colman. The subject will be "Egypt," and the lecture will be copiously illustrated by limelight views.

The Dowdeswell Galleries, 160, New Bond Street.—The Dowdeswell Galleries are especially attractive at the present time. Munkacsy's wonderful picture, "Ecce Homo," is on view, and should certainly be seen by any one interested in pictures. The dramatic grouping of the excited Jewish crowd, the contrast of the self-contained Roman officials, who environ the central spiritualised figure of Christ, form a most striking and impressive picture which it is not easy to forget. The look of execration on the countenances of the crowd is so life like that one seems almost to hear the words, "Crucify Him, crucify Him." The figures are life-size, the grouping is magnificent, and the whole interpretation of the subject is so powerful that it is not easy to forget this remarkable picture by the well-known Hungarian painter. In another portion of the galleries some charming scenes of the Down Country are being shown by Mr. Thorne Waite. They breathe with the happy peaceful spirit of our pastural land.

### For Reading to the Sick.

### "HIS TENDER MERCIES."

**Verses.**

Does not His mercy to His own exceed

The holiest form of kindness ever known

To human love! That spirit best can read

His love which scans the fulness of its own.

Ye fearful saints, fresh courage take!
The clouds ye so much dread,
Are big with mercy, and shall break
In blessings on your head. —*Cowper.*

Teach me to feel another's woe,
To hide the fault I see!
That mercy I to others show,
That mercy show to me! —*Pope.*

Such mercy He by His most holy reede,
Unto us taught, and to approve it trew
Ensampled it by His most righteous deede,
Showing us mercy, miserable crew!
That we the like should to the wretches shew,
And love our brethren. —*Spenser.*

On Thy compassion I repose
In weakness and distress:
I will not ask for greater ease,
Lest I should love Thee less.
Oh! 'tis a blessed thing for me
To need Thy tenderness!
—*A. L. Waring.*

**Reading.**

His mercy endureth for ever!

Blessed are the merciful, for they shall obtain mercy.—*Matt.* v 7.

It is of the Lord's mercies that we are not consumed, because His compassions fail not. They are new every morning.—*Jam.* iii. 22, 23.

If the Most High shall not multiply His mercies, the world would not continue with them that inherit it.

His tender mercies are over all His works.—*Ps.* cxlv. 9.

How shalt thou hope for mercy, rendering none?—*Shakespeare.*

We know not how greatly we are every day protected by God's heavenly might; we know not how He has already succoured us, how He has curbed the power of the enemy; we cannot tell from what bodily affliction, from what mental anguishes, from what fearful falls He has actually kept us. Let us then fear nothing but separation from Him; let us buckle on and brighten up our Christian armour that we may be able "to stand against all the fiery darts of the devil." Let us believe in the greatness of our redemption, in the presence of Christ, in the aid of the Holy Spirit, in the treasures of grace which are opened to us. Let us see what a safe and blessed thing it is to be on God's side; there can be in the universe no real danger for the "man who trusteth in Him." Let us watch our wavering wills, let us sit often in meditation beneath the cross of our only Lord. Let us thus resist the devil, and as he fleeth from us, "Behold, angels shall come and minister unto us." —*Bishop Wilberforce.*

Every day seems to shed the light which God gives with it on some fresh proof of His tender mercy; and every such proof of His goodness calls for the voice of a thankful spirit from my heart. I dread lest I, or any of us, should receive His blessings, whether earthly or heavenly, with a thankless spirit. Lord give us a due sense of all Thy mercies, "that our hearts may be unfeignedly thankful; and that we show forth Thy praise, not only with our lips, but in our lives; by giving up ourselves to Thy service, and by walking before Thee in holiness and true righteousness all the days of our lives."

### Notes and Queries.

**The contents of the Editor's Letter-box have now reached such unwieldy proportions that it has become necessary to establish a hard and fast rule regarding Answers to Correspondents. In future, all questions requiring replies will continue to be answered in this column without any fee. If an answer is required by letter, a fee of half-a-crown must be enclosed with the note containing the enquiry. We are always pleased to help our numerous correspondents to the fullest extent, and we can trust them to sympathise in the overwhelming amount of writing which makes the new rules a necessity. Every communication must be accompanied by the writer's name and address, otherwise it will receive no attention.**

*Hands and Feet.*

(129) I am going as probationer into a large hospital in a few weeks time. Would you kindly tell me what you consider the best preventative for bad hands and feet? Also (2) if I am expected to have anything in the way of instruments for the first month?—*B. B.*

Be careful to wash the feet daily, giving them plenty of friction with the towel when drying them, and then rub with dry alum. Continue to wear the heels of your shoes the height to which you are accustomed, having indiarubber put on them to prevent noise. The hands will not suffer if properly dried, and washed at the "toilette" with cold cream soap. (2) No.

*Training as Probationer.*

(130) Could you oblige me by telling me where to apply to obtain training as probationer?—*M. Dw. M.*

Apply to the matron of any hospital or infirmary that is recognized by the Local Government Board as a training school. The "Mirror" for April 3rd, 1897, "Hospital Nursing; Where and How to Train," gives a list of the principal London schools, and Honnor Morten's "How to Become a Nurse" does the same.

*A London Hospital.*

(131) Can you tell me if my going into a medical and surgical Home at Southampton for three years would afterwards prevent me from entering a London hospital?—*J. H. N.*

Certainly not, if you wished to enter the London hospital as a probationer afterwards, but it will not count in your training.

*Nurse-Companion.*

(132) I am desirous of becoming a nurse-companion to a lady for whom a highly-skilled nurse is not necessary. I have a thorough knowledge of physiology and the theory of nursing, also hold the St. John Ambulance Association "first aid" and "nursing" certificates, but have had little practical knowledge. Will you kindly tell me; (1) If I could obtain sufficient practical experience for this post in three or four months in a good nursing home? (2) Can you recommend such an institution? (3) About what sum is it usual to pay upon entry?—*Nurse-Companion.*

We should recommend your training for at least a year. Apply at one of the small special hospitals, such as, for instance, the Grosvenor Hospital for Women, Vincent Square, Westminster, where you would learn to attend to gynæcological cases. A nurse-companion with this knowledge ought to do very well. If you could spare two years you could get a certificate.

*Simple Ailments.*

(133) Can you kindly tell me of a book that describes simple ailments and their remedies? I want to send one out to British Central Africa.—*Nurse Blayney.*

"Helps in Sickness and to Health: Where to Go and What to Do," by Sir Henry C. Burdett, price 5s., from the Scientific Press, 28 & 29, Southampton Street, Strand.

*Homes for the Delicate.*

In answer to "Semper Paratus" we have received a large number of replies, some of which have been sent on direct to the querist. The Hon. Mrs. Fremantle, the Dean of Ripon's wife, writes to recommend Erith House, Torquay. She says that the house is in thorough sanitary order and that the charge is £1 1s. a week, and that the lady superintendent was a former "sister" at St. Thomas's. The institution is in need of funds, as old subscribers are dying off and new ones have not replaced them owing to the difficulty of making the home known. The home is for ladies in delicate health. Miss Violetta Fasulo, a trained nurse who has lived in Florence for many years, sent particulars of her nursing home in Via Rondivelli F. mezzannio, Florence. Nurses can be comfortably lodged and boarded at a small cost, and are helped in their business transactions. This home was started last November. We have written to one of Miss Fasulo's English references and have received a satisfactory reply.

(134) Can you tell me the price of binding THE HOSPITAL?—*Nurse Lena.*

The price is 2s. 6d. a volume. Write to the Manager of THE HOSPITAL.

*Water Cure Establishment.*

(135) Can you tell me the most perfect establishment in England in connection with the water cure for patients suffering from diseases of the nerves? Can you give me the information at once privately?—*J. M.*

It is impossible to advise an individual establishment. The place to which a patient should be sent depends much on the nature of the case, and should be decided by the medical man in charge. Our rule is that all queries requiring a private answer must be accompanied by a fee—2s. 6d.

### ANSWERS REQUESTED.

*Commode.*

(136) Would you be good enough to tell whether you know of any improvement on the "commode" commonly used in private houses? I need a small and a light one to remove for a patient unable to leave her bedroom—a very small bedroom. I would be grateful also to know the place from which to order it.—*W.*

